# Trace amounts of African swine fever virus DNA detected in insects collected from an infected pig farm in Estonia

**DOI:** 10.1002/vms3.200

**Published:** 2019-09-27

**Authors:** Reet Herm, Lea Tummeleht, Margret Jürison, Annika Vilem, Arvo Viltrop

**Affiliations:** ^1^ Equine Clinic Chair of Clinical Veterinary Medicine Estonian University of Life Sciences Tartu Estonia; ^2^ Chair of Veterinary Bio‐ and Population Medicine Estonian University of Life Sciences Tartu Estonia; ^3^ Chair of Plant Health Estonian University of Life Sciences Tartu Estonia; ^4^ Department of Molecular Analysis Veterinary and Food Laboratory Tartu Estonia

**Keywords:** mechanical vectors, real‐time PCR, transmission routes

## Abstract

**Background:**

African swine fever (ASF), a severe multi‐systemic disease in pigs, was introduced into Estonia in 2014. The majority of outbreaks have occurred during the summer months. Given that ASFV is transmitted in a sylvatic cycle that includes the transmission by African soft ticks and that mechanical transmission by flying insects was shown, transmission by other arthropod vectors need to be considered.

**Objectives:**

Here, we report the results of a pilot study on flying insects caught on an outbreak farm during epidemiological investigations.

**Methods:**

In brief, 15 different insect species (flies and mosquitoes) were collected by random catch using an aerial net. Nucleic acids derived from these samples or their pools were tested for African swine fever virus (ASFV) DNA by real‐time PCR.

**Results and Conclusions:**

Viral DNA was detected in small quantities in two samples from flies and mosquitoes. Given the slow spread of virus within the farm, the impact of these findings seems rather low, but a role in local transmission cannot be ruled out. However, given the very low number of insects sampled, and taken into the account that viral isolation was not performed and insects outside the farm were not investigated, future investigations are needed to assess the true impact of insects as mechanical vectors.

## Graphical abstract

Flying insects collected in a pig farm affected by African swine fever were analysed for the presence of viral DNA using real‐time PCR method. Trace amounts of African swine fever DNA was discovered in a small number of insects.

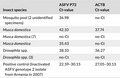

## 1 INTRODUCTION

African swine fever (ASF) is a severe contagious viral disease of domestic pigs and Eurasian wild boars (*Sus scrofa*). African swine fever virus (ASFV) is endemic in sub‐Saharan Africa, having a wildlife reservoir in resistant African species of wild *Suidae* that, although infected, remain asymptomatic, and in soft ticks of the *Ornithodoros* genus (Costard, Mur, Lubroth, Sanchez‐Vizcaino, & Pfeiffer, 2013). The most recent epidemic in Europe caused by the ASFV genotype 2 began in 2007 from Caucasus, spreading to the Russian Federation in 2007, where it became endemic (Gogin, Gerasimov, Malogolovkin, & Kolbasov, 2013), to Ukraine in 2012 (OIE, 2012) and Belarus in 2013 (OIE, 2015). It reached the European Union in 2014, when it was reported in wild boars in Lithuania, Poland, Latvia and Estonia (Guinat et al., 2016; Sánchez‐Vizcaíno, Mur, Gomes‐Villamandos, & Carrasco, 2015). To date, ASF has also been reported in Moldova, the Czech Republic, Romania, Bulgaria, Belgium (Frant, Woźniakowski, & Pejsak, 2017) and China (Zhou et al., 2018).

ASFV transmission can occur through animal contact, contaminated feed and fomites, as well as via arthropods (Costard et al., 2013). The role of ticks in ASFV transmission has also been examined in Europe (Portugal, Spain and Russia). In the Iberian Peninsula, ASFV is transmitted by ticks belonging to *O. erraticus* complex (Boinas et al., 2014). Certain *Ornithodoros* species (*O. erraticus* comple*x*) have been found in the Caucasus and in parts of Southern Europe (Manzano‐Román, Díaz‐Martín, de la Fuente, & Pérez‐Sánchez, 2012). One experimental study demonstrated the presence of ASF virus in *O. erraticus* for up to 12 weeks, but so far, field studies in Germany have failed to demonstrate *O. erraticus* contact in wild boars (Pietschmann et al., 2016). In the Russian Federation, no evidence of *Ornithodoros* involvement in the epidemic has been found (Gogin et al., 2013). Currently, no studies have shown evidence of ASF replication in the hard ticks prevalent in Europe (de Carvalho Ferreira et al., 2014). Field studies have shown a lack of virus in hard ticks, and under experimental conditions, hard ticks have failed to transmit the virus (Frant et al., 2017). In the absence of a tick vector, other arthropods have been considered. Flies have vector potential for several pathogens (Blunt et al., 2011; Förster et al., 2007), and various other biting insects, such as mosquitoes (Baldacchino et al., 2013; Maggi, ) and hog lice (Hornok et al., 2010), are known or suspected to transmit viral, bacterial and rickettsial infections. One experimental study has shown successful mechanical transmission of ASFV to pigs by stable flies (*Stomoxys calcitrans)* up to 24 hr after ingesting viraemic blood and subsequently feeding on pigs. ASFV titres were present in some flies up to 2 days post‐ingestion, making viral transmission via flies within this time period feasible (Mellor, Kitching, & Wilkinson, 1987). It was recently experimentally demonstrated that ASFV remains infectious in the stomach of some stable flies for up to 12 hr, and ASFV DNA was found on the flies’ bodies up to 72 hr after feeding (Olesen, Hansen, et al., 2018b). A subsequent experiment showed that after feeding on viraemic blood, flies then ingested by healthy pigs caused ASFV infection (Olesen, Lohse, et al., 2018a). There was no evidence of ASFV in flies in infected farms in Lithuania in 2014 (EC, 2014).

In Estonia, ASF was reported in 27 domestic pig farms during 2015–2017 (Nurmoja et al., 2018). Epidemiological investigations of these outbreaks suggest that the virus was most likely introduced into the herds through indirect transmission, such as by contaminated fomites, including vehicles, clothing, and contaminated feed or bedding material. ASF outbreaks in domestic pig farms in Estonia follow a seasonal pattern, occurring from July to September (Nurmoja et al., 2018). More rapid spread of the virus among wild boar also occurs in summer months (Nurmoja et al., 2018). This seasonal pattern of domestic outbreaks and spread in wild boar suggests a possible role for flying insects in viral spreading. Several vector‐borne diseases follow a similar trend, such as West Nile virus (Hayes et al., 2005) and Lyme borreliosis in humans (Jaenson & Lindgren, 2006). Based on the correlation between vector abundance and disease occurrence, flies have been suspected to be mechanical vectors for viral diseases, namely, *Musca domestica* in human shigellosis (Farag et al., 2013), and *S. calcitrans* in bovine lumpy skin disease (Kahana‐Sutin, Klement, Lensky, & Gottlieb, 2017).

To test this hypothesis, flying insects were collected from one infected farm and investigated for the presence of ASFV DNA by real‐time PCR.

## 2 CASE REPORT

### 2.1 Case details

**Figure 1:**
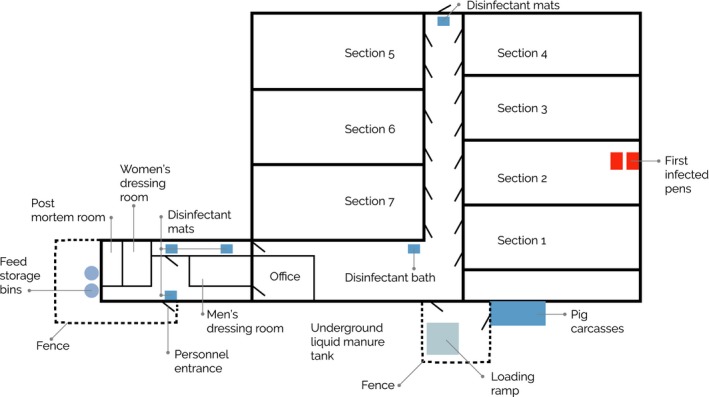
Schematic of the pig farm with the first infected pens highlighted in Section 2

In August 2016, an ASF outbreak was investigated in a fattening farm in the western part of Estonia. The outbreak occurred in one of the six isolated units of the farm building, each containing approximately 500 pigs (Figure 1). On 16th of August, decreased appetite was noticed in several pigs in two pens in Section 2. On 18th of August, one of the pigs in these pens was noticed to have cyanotic ears. The pig died shortly thereafter. The number of inappetent pigs increased. On the next day, two more pigs died in the same pens. Antimicrobial treatment was initiated in the diseased pens with the suspicion of bacterial infection. Between 19th and 22nd August, an additional 31 pigs died in the affected pens, bringing the total number to 34.

### 2.2 Postmortem findings and laboratory analyses

Fifteen pig cadavers were necropsied on the farm by the attending veterinarian. Postmortem findings included cyanotic ears, spleno‐ and hepatomegaly, petechiae on kidneys and around cardiac valves, and generalized lymph node enlargement. A small amount of serous fluid was found in the abdominal cavity. African swine fever was suspected, and the district veterinary office of the Veterinary and Food Board (the state veterinary authority) was notified. Later on the same day, the farm veterinarian, together with the local animal health surveillance officer, necropsied an additional nine pigs. Blood and tissue samples were sent to the national ASF diagnostic laboratory (Veterinary and Food Laboratory) for ASFV testing. On 23rd of August, ASFV was confirmed in pigs by real‐time PCR and antibody ELISA from serum and tissue samples. On 24th of August, additional animals of the farm were killed on site. Before culling, additional blood samples (*n* = 18) were collected from pigs in five other farm units targeting animals with any suspicion signs of disease. All samples collected from other units tested negative by both PCR and ELISA.

### 2.3 Insect sampling and DNA analysis

On 24th of August, the outbreak was investigated by epidemiologists from Estonian University of Life Sciences. Flying insects have been considered one possible means of spreading the infection within and between farms. Therefore, it was decided to investigate whether ASFV‐DNA could be detected in flying insects caught in a naturally infected farm. If successful, the intention was to use this method in future outbreak investigations. Random flying insects in close contact with pigs in the affected unit were caught using an aerial net. Insects were killed using chloroform and stored at −20°C until species identification and DNA extraction.

Insects (*n* = 15) were identified morphologically using identification keys, and those identified included haematophagous *Culicidae spp* (*n* = 2), *M. domestica* (*n* = 9) and *Drosophila spp.* (*n* = 4).

For DNA extraction, whole individual insects were homogenized and DNA was extracted using an RTP DNA/RNA Virus Mini Kit, (Stratec, Germany) according to the manufacturer's instructions. The two mosquito specimens were pooled and processed together.

DNA samples were analyzed by real‐time PCR methods using ASFV p72 gene‐targeting forward and reverse primers and TaqMan probes, as described by Tignon et al. (2011). For an endogenous control in the assay, swine beta‐actin (ACTB) gene detection was included in the analysis with forward) and reverse primers and probes targeting the 114 bp‐region of the gene (Duvigneau, Hartl, Groiss, & Gemeiner, 2005). Real‐time PCR was performed using the commercially available 5xHOT FIREPol Probe qPCR Mix kit (Solis BioDyne) in a total volume of 20 µl. Briefly, 7 µl DNAse RNase free water, 4 µl 5x HOT FIREPol Probe qPCR Mix, 0.8 µl each of forward and reverse primers targeting the ASF p72 and swine beta‐actin gene at a final concentration of 0.4 µM and 0.4 µl each of probe at a final concentration of 0.2 µM were pooled as a master mix. Finally, a 5 µl of aliquot of DNA extracted from sample was added to 15 µl of PCR master mix. The cycling protocol was as follows: 1 cycle of 95ºC for 15 min followed by 45 cycles consisting of denaturation for 20 s at 95°C and annealing for 1 min at 60°C. Threshold cycle (Ct) values less than 40 were considered positive.

### 2.4 Viral sequencing

Sequencing of the B602L gene to determine the CVR (central variable region) variant was performed in all ASF positive samples using the standard operating procedure provided by the European Union Reference Laboratory of ASF (CISA‐INIA) with minor modification regarding the PCR amplification protocol. The B602L gene was amplified with forward and reverse primers as described by Gallardo et al. (2011). PCR was performed using a commercially available AmpliTaq Gold™ DNA Polymerase with Gold Buffer and MgCl_2_ PCR kit (Applied Biosystems) in a total volume of 25 µl. Briefly, 15.875 µl DNA‐RNA free water, 2.5 µl 10x PCR buffer, 2.5 µl MgCl_2_, and 0.5 µl dNTP (Applied Biosystems) in a final concentration of 0.2 mM, 0.75 µl both of forward and reverse primers at a final concentration of 0.3 mM and 0.125 µl AmpliTaq Gold Polymerase at a final concentration of 0.625 U was pooled as a master mix. Finally, 2 µl ASF positive DNA was added to 23 µl PCR master mix. Cycling conditions were as follows: 1 cycle of 95°C for 10 min followed by 40 cycles consisting of denaturation for 30 s at 95°C, annealing for 30 s at 55°C and elongation for 1 min at 72°C, and finally 1 cycle of 72°C for 10 min. Amplified PCR products were visualized using 2% agarose gels.

Sample sequencing was performed at the University of Tartu, Institute of Genomics. PCR products were cleaned and sequenced with Applied Biosystems ® 3130xl Genetic Analyzer by a two‐directional procedure. Forward and reverse sequences were aligned using MEGA7 software (Kumar, Stecher, & Tamura, 2016) and BioEdit v7.2.5 software (Hall, 1999) to generate single consensus sequences and to correct mismatches. Acquired sequences were compared against nucleotide sequences available in GenBank using BLASTn (nucleotide Basic Local Alignment Search Tool).

## 3 RESULTS AND DISCUSSION

Two of 13 flies (one housefly and one drosophila), as well as the mosquito pool of two individuals, tested positive for ASFV‐DNA. Results of the beta‐actin qPCR were inconsistent, being positive in 2 fly samples (one ASFV qPCR positive, one negative) but negative in the mosquito pool (see Table 1 for reference). Overall, the Ct values were high, indicating only small amounts of target DNA in the samples. It was possible to obtain DNA sequences from three of the positive samples, which were subsequently determined as genotype 2 CVR variant 1, which is the most common variant spreading in Estonia and the EU. Virus isolation from positive samples was not performed due to laboratory limitations.

**Table 1:** Ct‐values of viral DNA from collected insect samples analysed by real‐time assay of the C‐terminal end of the ASFV p72 gene

Insect species	ASFV P72 Ct‐value	ACTB Ct‐value
Mosquito pool (2 unidentified specimens)	36.98	no‐Ct
*Musca domestica*	42.10	37.74
*Musca domestica* (7)	no‐Ct	no‐Ct
*Musca domestica*	35.43	no‐Ct
*Drosophila* spp*.*	38.10	36.27
*Drosophila* spp. (3)	no‐Ct	no‐Ct
Positive control (Inactivated ASFV genotype 2 isolate from Armenia in 2007)	22.59–30.15	27.05–30.13

Note.

A target 114 bp region of the swine ACTB gene was included in the assay as an endogenous control. Ct values up to 40 were considered positive.

Abbreviations: ACTB, beta‐actin Ct, cycle threshold.

It was previously shown that blood‐sucking flies, such as stable flies, can transmit ASFV. It has also been experimentally demonstrated that stable flies can transmit ASFV either by biting (Mellor et al., 1987) or through ingestion by pigs (Olesen, Lohse, et al., 2018a). The authors further proposed that although larger blood‐sucking insects, such as horse flies (*Tabanidae*), do not preferentially feed on pigs, they may transmit the virus through being eaten by pigs, and therefore, blood‐sucking flies may play a role in virus transmission within farms or, in the case of *Tabanidae*, over longer distances (Olesen, Lohse, et al., 2018a). All of the flies caught in our study belonged to non‐blood sucking species. *M. domestica* and *Drosophila spp.* feed on decaying matter but could potentially carry the virus on their bodies (Merritt, Courtney, & Keiper, 2009)*.* To the authors’ knowledge, no information exists about transmission of ASFV by these insect species. *Drosophila* can either accidentally be ingested by pigs or they may contaminate pig feeding sources. Although live virus isolation was not attempted, the small amount of DNA material present, as indicated by high Ct values, is an indication of low viral load.

The Ct value of 42.10 in one tested *M. domestica* may indicate probe degradation. Then again, all samples were tested in duplicate, and parallel sample of the aforementioned Ct value of 42.10 resulted in no Ct value. However, this particular *M.domestica* was ACTB gene positive, indicating contact with pigs.

Contamination cannot be completely ruled out, especially considering that after catching, the insects were kept together during storage. The risk of contamination during DNA extraction and qPCR analysis was reduced by using negative controls and by closing all the test tubes prior to adding the positive control.

It is also worth noting that not all pigs in the unit were infected, and the virus did not spread to other units, despite the abundance of insects and the lack of restrictions to their flight to other parts of the building. The authors propose that the DNA finding on the insects most likely represents a general environmental contamination with the ASF virus. Alternatively, ASFV positive insects in this study did not carry live virus long enough to transmit it.

The study has several limitations. The sample size is too small to draw any conclusions regarding the sampled arthropods as possible ASFV vectors. The use of an aerial net combined with a relatively inexperienced catcher resulted in a small yield of insects. Another trapping method, such as a combination of sticky traps and aerial interception traps (Epsky, Morrill, & Mankin, 2008) would have caught a larger number of flying arthropods, but this was not deemed practical during an outbreak where the catching had to be performed in a limited time, and decontaminating the catching tools at the exit of the infected premises was required. Sampling was performed to determine if it was at all possible to identify ASFV DNA in flying arthropods under field conditions; therefore, other units of the building were not sampled. More thorough studies were planned, pending the preliminary results; however, in two subsequent outbreaks, sampling was not plausible as there were very few flying insects within the buildings due to effective control measures at these farms. No further studies were conducted as these were the last outbreaks in domestic pigs that Estonia has experienced since then.

The small sample size and lack of blood sucking flies leaves the study results open to discussion. The results of this study suggest that several insect species may be contaminated with (or possibly carrying) the virus but we were unable to establish that they had any significant role in transmission of ASFV within the farm. Further studies (particularly field studies) are needed to explore that possibility.

## CONFLICT OF INTEREST

The authors declare no conflict of interest.

## ETHICAL STATEMENT

The authors confirm that the ethical policies of the journal, as noted on the journal's author guidelines page, have been adhered to. No ethical approval was required as no animal testing was performed.
